# Interleukin-1 receptor antagonist (IL-1Ra) is more effective in suppressing cytokine-induced catabolism in cartilage-synovium co-culture than in cartilage monoculture

**DOI:** 10.1186/s13075-019-2003-y

**Published:** 2019-11-13

**Authors:** Shikhar Mehta, Sumayyah Akhtar, Ryan M. Porter, Patrik Önnerfjord, Ambika G. Bajpayee

**Affiliations:** 1 Department of Bioengineering, Northeastern University, 805 Columbus Avenue, Boston, MA 02120 USA; 2 Department of Biochemistry, Northeastern University, 805 Columbus Avenue, Boston, MA 02120 USA; 3 Departments of Internal Medicine and Orthopaedic Surgery, University of Arkansas for Medical Sciences, 4301 W. Markham, Little Rock, AR 72205 USA; 4 Department of Rheumatology, Lund University, BMC-C12 Klinikgatan 28, 222 42 Lund, Sweden; 5 Department of Mechanical Engineering, Northeastern University, 805 Columbus Avenue, Boston, MA 02120 USA

**Keywords:** IL-1Ra, IL-1, Cartilage-synovium co-culture, Crosstalk, Sustained dose, Nitric oxide

## Abstract

**Background:**

Most in vitro studies of potential osteoarthritis (OA) therapies have used cartilage monocultures, even though synovium is a key player in mediating joint inflammation and, thereby, cartilage degeneration. In the case of interleukin-1 (IL-1) inhibition using its receptor antagonist (IL-1Ra), like chondrocytes, synoviocytes also express IL-1 receptors that influence intra-articular IL-1 signaling and IL-1Ra efficacy. The short residence time of IL-1Ra after intra-articular injection requires the application of frequent dosing, which is clinically impractical and comes with increased risk of infection; these limitations motivate the development of effective drug delivery strategies that can maintain sustained intra-articular IL-1Ra concentrations with only a single injection. The goals of this study were to assess how the presence of synovium in IL-1-challenged cartilage-synovium co-culture impacts the time-dependent biological response of single and sustained doses of IL-1Ra, and to understand the mechanisms underlying any co-culture effects.

**Methods:**

Bovine cartilage explants with or without synovium were treated with IL-1α followed by single or multiple doses of IL-1Ra. Effects of IL-1Ra in rescuing IL-1α-induced catabolism in cartilage monoculture and cartilage-synovium co-culture were assessed by measuring loss of glycosaminoglycans (GAGs) and collagen using DMMB (dimethyl-methylene blue) and hydroxyproline assays, respectively, nitric oxide (NO) release using Griess assay, cell viability by fluorescence staining, metabolic activity using Alamar blue, and proteoglycan biosynthesis by radiolabel incorporation. Day 2 conditioned media from mono and co-cultures were analyzed by mass spectrometry and cytokine array to identify proteins unique to co-culture that contribute to biological crosstalk.

**Results:**

A single dose of IL-1Ra was ineffective, and a sustained dose was necessary to significantly suppress IL-1α-induced catabolism as observed by enhanced suppression of GAG and collagen loss, NO synthesis, rescue of chondrocyte metabolism, viability, and GAG biosynthesis rates. The synovium exhibited a protective role as the effects of single-dose IL-1Ra were significantly enhanced in cartilage-synovium co-culture and were accompanied by release of anti-catabolic factors IL-4, carbonic anhydrase-3, and matrilin-3. A total of 26 unique proteins were identified in conditioned media from co-cultures, while expression levels of many additional proteins important to cartilage homeostasis were altered in co-culture compared to monocultures; principal component analysis revealed distinct clustering between co-culture and cartilage and synovium monocultures, thereby confirming significant crosstalk.

**Conclusions:**

IL-1Ra suppresses cytokine-induced catabolism in cartilage more effectively in the presence of synovium, which was associated with endogenous production of anti-catabolic factors. Biological crosstalk between cartilage and synovium is significant; thus, their co-cultures should better model the intra-articular actions of potential OA therapeutics. Additionally, chondroprotective effects of IL-1Ra require sustained drug levels, underscoring the need for developing drug delivery strategies to enhance its joint residence time following a single intra-articular injection.

## Background

Interleukin-1 (IL-1) is a pro-inflammatory cytokine elevated after traumatic injury that stimulates cartilage degradation, suppresses matrix biosynthesis, and induces chondrocyte apoptosis, mechanisms associated with progression to post-traumatic osteoarthritis (PTOA) [1]. PTOA accounts for 12% of all OA cases and primarily affects younger and more active populations [2, 3]. IL-1 stimulates pro-inflammatory/catabolic activities by binding with the widely expressed IL-1 receptor type I (IL-1R1), forming a high-affinity complex with the IL-1R accessory protein (IL-1RAcp) that activates multiple intra-cellular signal transduction pathways, such as nuclear factor kappa-light-chain-enhancer of activated B cells (NF-κB) [4]. IL-1Ra (MW ~ 17.6 kDa), a receptor antagonist of IL-1, can competitively bind with IL-1R1 thereby blocking cell activation by the cytokine [5]. IL-1Ra has thus been considered as a promising disease-modifying OA drug (DMOAD) based on encouraging in vitro and pre-clinical in vivo data from experimental arthritis and osteoarthritis models [6–9].

Most in vitro studies of cartilage catabolism and potential protective therapies have used cartilage monocultures, even though OA is a disease of the entire joint involving interactions between multiple tissues. Synovium, in particular, is known to be a key player in mediating joint inflammation especially in diseased joints through cellular infiltration (CD4^+^ lymphocytes and CD68^+^ macrophages), angiogenesis (VEGF production), release of inflammatory mediators (IL-1, TNFα, IL-6, IL-8), and formation of nociceptive fibers [10]. Recent studies using magnetic resonance imaging (MRI) have demonstrated strong correlations between presence of synovitis (synovial inflammation and thickening) in early OA, pain, and disease progression in the joint [11, 12]. In fact, diagnosis of synovitis may provide an initial indication of impending OA and facilitate early intervention, when disease-modifying drugs like IL-1Ra can be most effective at targeting inflammatory processes. Like chondrocytes, synoviocytes also express IL-1R1, resulting in significant crosstalk between these populations that determines the overall biological response to IL-1 and to its inhibitors like IL-1Ra. In vitro studies of IL-1 antagonism might therefore require cartilage-synovium co-culture models for assessing disease pathogenesis, progression, and response to therapeutics.

Despite promising pre-clinical studies, clinical translation of IL-1Ra for OA treatment remains a challenge, which has been partly attributed to its short joint residence time and its lack of ability to co-target multiple joint tissues [13]. Most in vitro culture experiments and animal studies have used frequent doses to maintain sustained drug concentrations [6–9], which is clinically impractical due to patient discomfort and inconvenience; moreover, multiple intra-articular injections come with increased risk of joint infection and septic arthritis [14]. Therefore, there is a need for development of effective drug delivery strategies that can maintain sustained IL-1Ra concentrations for several weeks inside the joint and co-target multiple joint tissues following a single intra-articular (IA) injection. To that end, it is critical to understand the dynamics of a sustained drug dose and compare with those of a single dose of IL-1Ra in rescuing cytokine-induced catabolism.

Here we compare the time-dependent bio-activity of a single dose (mimicking a single injection in vivo) with that of multiple doses (mimicking sustained drug concentration that an effective drug delivery system would enable with a single IA injection) of IL-1Ra in both monoculture of cartilage and co-culture of cartilage and synovium explants in order to investigate the role of cartilage-synovium crosstalk. The goals of this study were (1) to assess how the presence of synovium in IL-1-challenged cartilage-synovium co-culture impacts the effectiveness of single and sustained doses of IL-1Ra compared to that in cartilage monoculture and (2) to understand the underlying mechanisms of interaction between co-culture and IL-1Ra treatment by identifying unique proteins that contribute to cartilage-synovium crosstalk.

## Methods

### Materials

Dulbecco’s modified Eagle’s medium (DMEM) was from Cellgro (Manassas, VA). HEPES, 100× non-essential amino acids (NEAA), and 100× insulin-transferrin-selenium (ITS) were purchased from Gibco (Carlsbad, CA). Ascorbic acid and l-proline were from Fisher Bioreagents (Pittsburgh, PA). Human recombinant IL-1α and human recombinant IL-1Ra were from PeproTech (Rocky Hill, NJ). Radiolabeled ^35^S-sulfate was from PerkinElmer (Waltham, MA). Proteinase K was purchased from Roche Diagnostics (Risch-Rotkreuz, Switzerland). Dermal punches were purchased from Moore Medical (Farmington, CT). Tissue culture well plates were from Cellgro (Manassas, VA). Additional reagents were from Sigma-Aldrich (St. Louis, MO) where not otherwise noted.

### Bovine cartilage and synovial joint capsule harvest

**Fig. 1:**
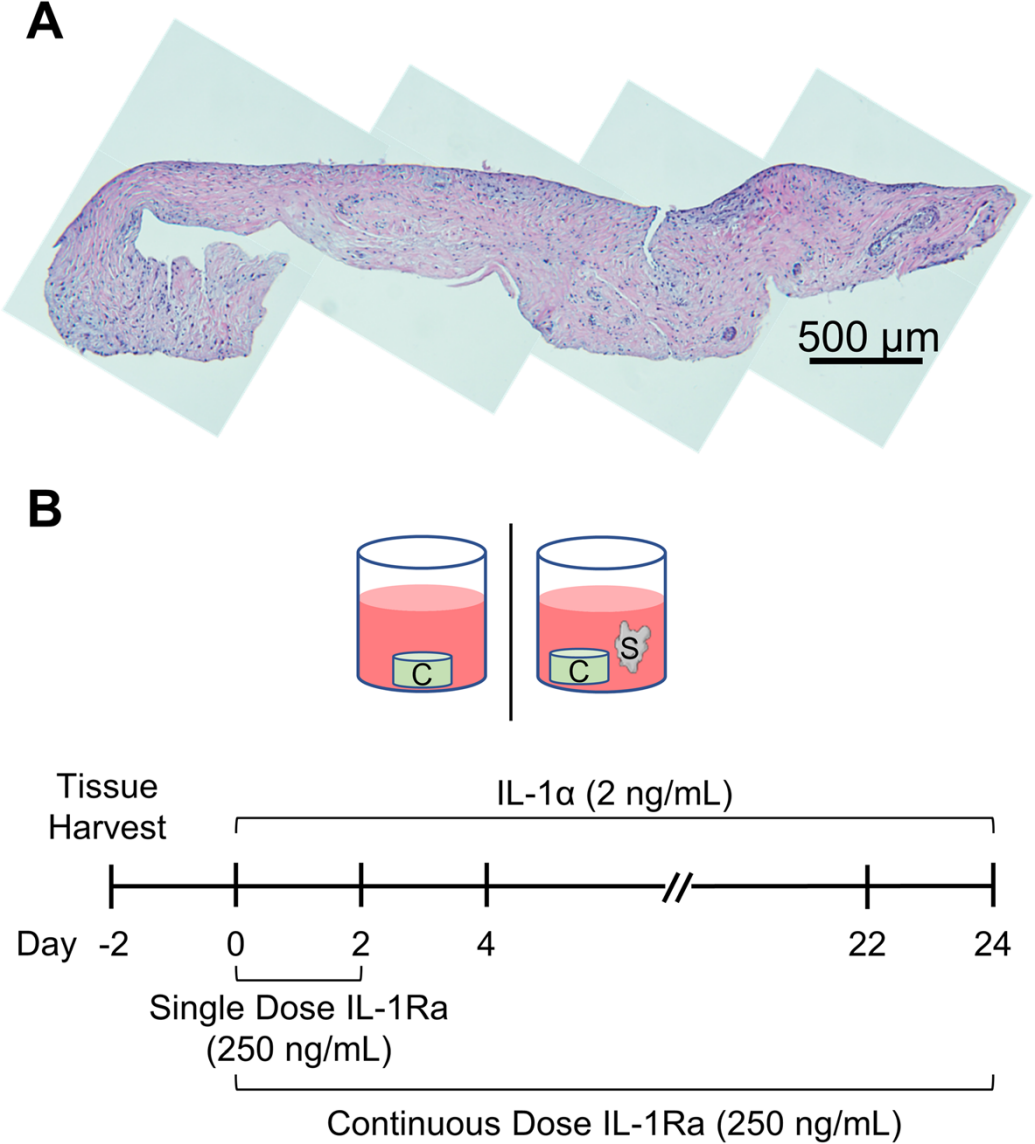
**a** H&E-stained section of bovine synovium harvested proximal to the patella. Tissue was cut to 25 ± 3 mg pieces containing a single intimal layer of synovium and no vasculature. Multiple images taken at × 10 magnification and stitched together to show the entire length of the tissue. **b** Experimental design. Cartilage tissue samples were cultured individually or in co-incubation with synovium tissue in serum-free media for 24 days. IL-1α was replenished every 2 days; IL-1Ra was replenished every 2 days in a continuous-dose condition while it was removed after day 2 in a single-dose condition

Cartilage disks (3 mm diameter, 10 ± 1 mg) were harvested from femoropatellar grooves of 1–2-week-old bovine calf knees (Research 87, Boylston, MA) using a 3-mm diameter dermal punch and sliced to obtain the top 1-mm disk with an intact superficial zone 10.1016/j.jbiomech.2018.06.012 [15]. Synovial joint capsule was harvested from the medial and lateral sides to the patella of the same animal and cut to 25 ± 3 mg pieces using a pair of sterile surgical scissors [9, 16]. This tissue was about 0.5 to 1 mm thick containing a single layer of synovium as shown in Fig. 1a. Tissue explants for all treatment conditions were matched for depth and location to prevent any bias. Explants were equilibrated individually in serum-free medium (low-glucose DMEM) for 2 days at 37 °C and 5% CO_2_ prior to co-incubation and any treatment. Media was supplemented with HEPES buffer, ITS, NEAA, and antibiotic antimycotic (100×) per manufacturer’s recommendations at 1% (V/V) each. Media was further supplemented with proline (11.5 mg/mL stock) and ascorbic acid (5 mg/mL stock) at 0.4% (V/V) each.

### Treatment of bovine tissues with exogenous IL-1α and human recombinant IL-1Ra

Cartilage explants were cultured individually (C) or in combination with synovium (C+S) with or without 2 ng/mL IL-1α over a period of 24 days in the presence of the following conditions: (i) a single dose of 250 ng/mL human recombinant IL-1Ra and (ii) a continuous dose of 250 ng/mL IL-1Ra (Fig. 1b). Medium was changed every 2 days and IL-1α was replenished. Single-dose IL-1Ra-treated explants were subjected to IL-1Ra for only the first 2 days; following medium changes did not contain IL-1Ra, thereby simulating a single intra-articular injection of IL-1Ra in vivo [17]. Continuous dose IL-1Ra condition was replenished with IL-1Ra throughout the culture duration. IL-1α concentration was chosen as it represents a moderately aggressive cytokine treatment [1]. IL-1Ra concentration was chosen based on in vitro studies that show 100-fold higher IL-1Ra than IL-1 is effective in blocking IL-1 activity [9].

### Tissue sulfated glycosaminoglycan (sGAG) and collagen loss to medium

After 24 days of culture, cartilage and synovium explants were weighed and then digested with proteinase K. Cumulative release of sGAG to the culture supernatant and sGAG content in cartilage tissues were measured using the dimethyl-methylene blue (DMMB) dye binding assay [18]. Cumulative release of collagen to the culture supernatant and collagen content in cartilage and synovium tissues were measured using the hydroxyproline assay [19].

### Nitrite release from tissues to medium

Nitrite (NO_2_^−^) content was measured using the Griess assay as an indicator of nitric oxide (NO) release from tissues. Nitrites react with the Griess reagent to form an azo dye with an absorbance maximum at 540 nm wavelength. Equal volumes of Griess reagent and culture media collected every 2 days were mixed and incubated at room temperature for 15 min, and absorbance was measured using a plate reader (Microplate Reader, Biotek). Sodium nitrite was used as a standard.

### Cellular metabolism in bovine tissues

At days 8, 16, and 24 of culture, tissue explants were separated and individually incubated with media containing 1× resazurin sodium salt (Sigma, Alamar Blue assay) for 3 h in the dark at 37 °C and 5% CO_2_. Cell metabolic activity was estimated by measuring fluorescence at 530-nm excitation and 590-nm emission wavelengths.

### Chondrocyte viability in cartilage explants

Using previously described methods, 100–200-μm-thick slices were obtained from the center of cartilage disks in monoculture or co-culture from each treatment condition at days 8, 16, and 24 [17, 20]. Slices were then stained for 4–6 min in the absence of light with fluorescein diacetate (FDA; 4 mg/mL in DMSO) and propidium iodide (PI; 10 mg/mL in PBS). FDA stained viable cells green, while PI stained non-viable cells red. Cartilage slices were then washed with phosphate-buffered saline (PBS) and imaged under a Nikon fluorescence microscope using a  4× objective.

### sGAG biosynthesis rates in cartilage

After 14 days of culture, cartilage disks from (i) cartilage **(**C**)** and (ii) cartilage and synovium (C+S) groups treated with IL-1α and IL-1Ra were radiolabeled with 15 μCi/mL ^35^S-sulfate in fresh culture medium at 37 °C and 5% CO_2_ for 48 h (synovial capsule tissue was removed before the label). The disks then were washed to remove any unincorporated label, digested in proteinase K, and analyzed using liquid scintillation for radiolabeled newly synthesized sGAGs over 48 h.

### Cytokine analysis for tissue culture medium

A bovine cytokine array kit (RayBiotech) was used to qualitatively determine cytokine presence in day 2 conditioned media from all treatment conditions, per the manufacturer’s instructions. Results were quantified by calculating the mean spot density from the array using ImageJ, and results are shown in comparison to untreated control. Data presents average of two blots per treatment condition.

### Mass spectrometry

Discovery experiments (non-targeted mass spectrometry) were performed on day 2 conditioned media (48 h treatment) using a quadrupole Orbitrap benchtop mass spectrometer (QExactive) (Thermo Fisher Scientific, Waltham, WA) equipped with an Easy nano-LC 1000 system (Thermo Scientific, Waltham, MA). Separation was performed on 75 μm × 25 cm capillary columns (Acclaim PepmapTM RSLC, C18, 2 μm, 100 Å, Thermo Scientific, Waltham, WA). A spray voltage of + 2000 V was used with a heated ion transfer setting of 275 °C for desolvation. The on-line reversed-phase separation was performed on an Easy nano-LC 1000 system using a flow rate of 300 nl/min and a linear binary gradient from 3% solvent B for 60 min to 35% solvent B, then to 90% solvent B for 5 min and finally isocratic 90% solvent B for 5 min. An MS scan (400–1200 m/z) was recorded in the Orbitrap mass analyzer set at a resolution of 70,000 at 200 m/z, 1 × 10^6^ automatic gain control (AGC) target and 100 ms maximum ion injection time. The MS was followed by data-dependent high-energy collision-induced dissociation (HCD) MS/MS scans at a resolution of 15,000 on the 15 most intense multiply charged ions at 2 × 10^4^ intensity threshold, and dynamic exclusion enabled for 30 s.

### Mass spectrometry data analysis

Identification from discovery data was performed using the *Bos Taurus* taxonomy (23,969 sequences) setting of the UniProt database (UP_000009136 from 2017-10) with Proteome Discoverer 2.2 (version 2.2.0.388, Thermo Scientific). The processing workflow consisted of the following nodes: Spectrum Selector for spectra pre-processing (precursor mass range, 350–5000 Da; S/N Threshold, 1.5) and Sequest-HT search engine (enzyme, trypsin; max. missed cleavage sites, 2; peptide length range, 6–144 amino acids; precursor mass tolerance, 10 ppm; fragment mass tolerance, 0.02 Da; static modification, cysteine carbamidomethylation; dynamic modification, methionine oxidation, hydroxyproline, and pyroglutamic acid (N-terminal Glu to pyroglutamic acid), and percolator for peptide validation (FDR < 1% based on peptide *q* value)). Results were filtered to keep only the Master protein with at least one unique peptide, and protein grouping was allowed according to the parsimony principle. In addition, both proteins and peptide that were not identified in at least four samples were removed. Multiple peptides were measured for each protein using discovery-based proteomics and the intensity for each protein was determined by summing up peak area intensities from unique peptides for each protein representing the abundance of the protein in the explant medium. Peptide peak area intensities were quantified (without normalization) using a proprietary algorithm with feature detection and matching thereof, developed in Proteome Discoverer 2.2.

### Histology

Synovial tissue was fixed in 10% neutral buffered formalin before embedding in paraffin wax. Sections (5 μm) were collected orthogonal to the plane of the tissue sheet and stained with hematoxylin and eosin. Multiple images were obtained at  10× objective and stitched together to show the entire synovium section.

### Statistical analysis

For all explant studies, general linear mixed effects model was used with animals as a random variable, followed by Tukey’s honestly significant difference (Tukey’s HSD) test for comparisons between multiple treatment conditions. There was no effect on animal found and hence the data across animals were pooled. In general, *n* = 12–18 explants per treatment condition from 5 bovine joints (3 independent repeats) were used. Data are presented as mean values ± 95% confidence interval. *p* values less than 0.05 were considered statistically significant.

## Results

### A single dose of IL-1Ra was ineffective, and a sustained dose was necessary to significantly suppress IL-1α-induced GAG loss, collagen loss, and NO synthesis. Synovium exhibited a protective role as the effectiveness of single-dose IL-1Ra was enhanced in co-cultures

**Fig. 2:**
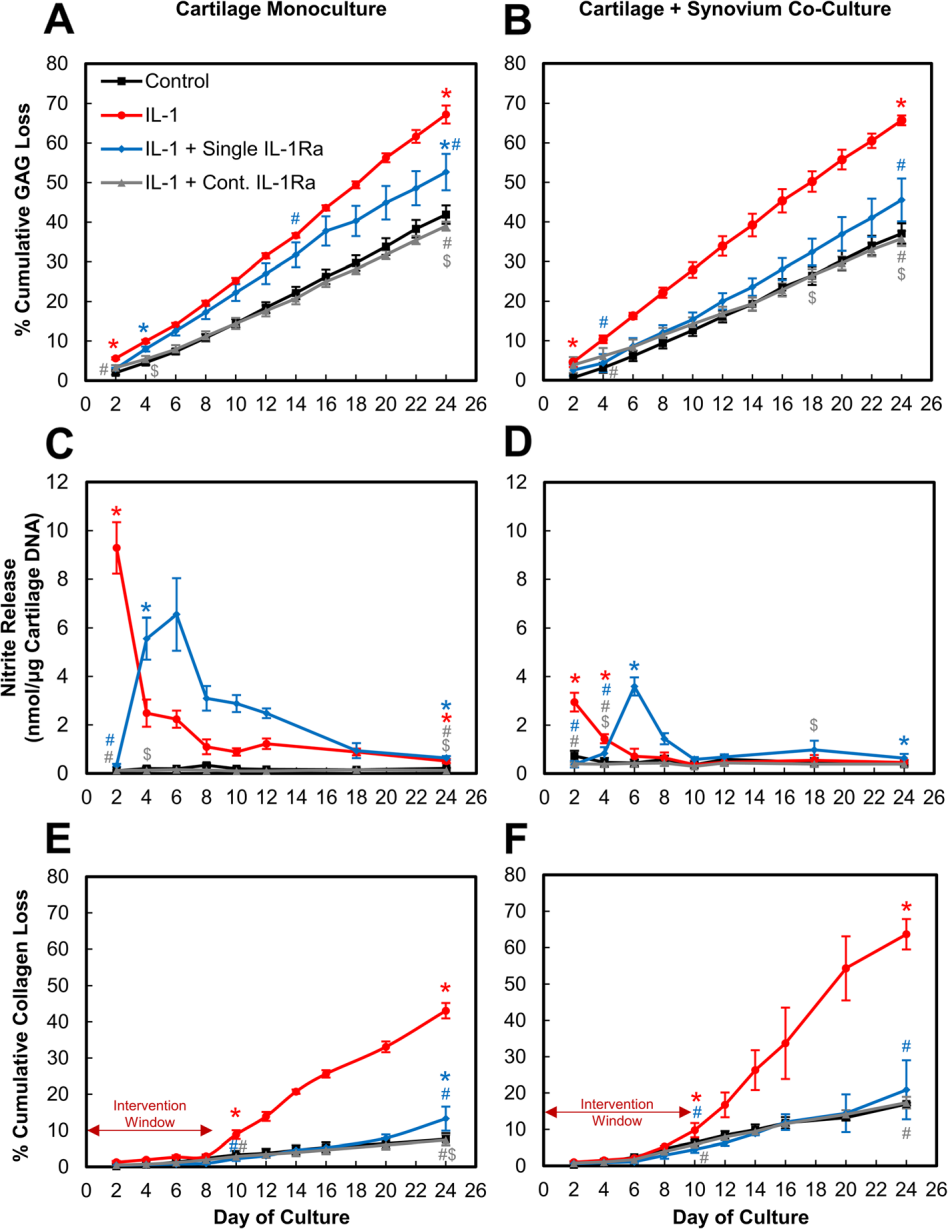
IL-1α-treated cultures administered with either a single or continuous (Cont.) dose of 250 ng/mL IL-1Ra for 24 days. Mean ± 95% confidence interval of cumulative sGAG release as percentage of total sGAG content measured every 2 days in **a** cartilage monoculture and **b** cartilage + synovium co-culture. Nitrite release in media of **c** cartilage monoculture and **d** cartilage + synovium co-culture. Cumulative collagen loss measured as percentage of total collagen content of tissues in **e** cartilage monoculture and **f** cartilage + synovium co-culture. Double arrow indicates intervention window during which therapy can be administered prior to loss of collagen from extracellular matrix. * vs untreated control, # vs IL-1, $ vs single-dose IL-1Ra, (*p* < 0.05). Statistical markers are color coordinated with all curves. All the data enclosed within similar markers are statistically significant

In cartilage monoculture, a single dose of 250 ng/mL IL-1Ra inhibited IL-1α-induced GAG loss only on day 2 (*p* < 0.0001), after which GAG loss remained statistically similar to IL-1-treated explants (Fig. 2a). A continuous dose, however, significantly suppressed IL-1-induced GAG loss throughout the 24 days of culture to levels similar to untreated controls. Beginning at day 4, a continuous dose of IL-1Ra resulted in significantly lower GAG loss compared to a single dose of IL-1Ra-treated explants (*p* < 0.0001). Similarly, in C+S co-culture, a single dose of IL-1Ra resulted in high GAG loss compared to the continuous dose but the difference became statistically significant only at later time points starting at day 18 (*p* < 0.046, Fig. 2b). Greater suppression of GAG loss was observed with a single dose of IL-1Ra in C+S co-culture compared to that in C monoculture that became statistically significant starting at day 8 (*p* < 0.045, Additional file 1: Figure S1 compares GAG loss data for C vs. C+S directly).

IL-1 is known to strongly stimulate nitric oxide (NO) production by the inducible nitric oxide synthase (iNOS) pathway in chondrocytes, contributing to inflammation and tissue destruction by enhancing production of matrix metalloproteinases (MMPs), inhibiting synthesis of collagen and proteoglycans, and promoting chondrocyte apoptosis [21, 22]. As expected, treatment with IL-1α significantly increased nitrite release in C monoculture and C+S co-culture compared to their respective untreated controls (*p* < 0.0001 through day 24 for C; *p* < 0.0001 through day 4 for C+S, Fig. 2c, d). Synovium monoculture did not produce significant nitrites in untreated condition (Additional file 2: Figure S2A). When challenged with IL-1, day 2 conditioned media from cartilage measured 76× higher levels of nitrites than that from synovium, suggesting that most nitrites are released by the cartilage cells (and in negligible amounts by synovium cells, thus the data presented is normalized by cartilage DNA. Nevertheless, even the small amounts of nitrite released from synovium make the values in C+S higher than in C. However, this does not imply that treatments in C+S performed worse than in C: when the data was normalized using total tissue DNA content, all control and continuous dosing curves from C and C+S collapsed to similar levels (Additional file 2: Figure S2B). Trends remained similar when the data was normalized by C+S DNA content or the total tissue weight (data not shown), instead of cartilage DNA content. Moreover, due to its short half-life, the biological effects of endogenously produced nitrites are expected to occur locally within the cartilage [22], as also supported by our data.

A single dose of IL-1Ra reduced IL-1α-induced nitrite release by 28× at day 2 in cartilage monoculture (*p* < 0.0001) after which the levels went back up and remained elevated through day 24 compared to untreated control (Fig. 2c). Continuous dosing of IL-1Ra, on the other hand, significantly reduced nitrite release to levels similar to control levels in monoculture, and they were significantly lower compared to both IL-1α (*p* < 0.0001) and a single dose of IL-1Ra (*p* < 0.0001)-treated conditions throughout the culture duration. In C+S co-culture, IL-1 significantly enhanced nitrite release compared to controls (*p* < 0.0001 until day 4), which was inhibited by a single dose of IL-1Ra until day 4 (*p* < 0.0028, Fig. 2d). Nitrite levels then spiked at day 6 and remained elevated throughout the culture period compared to untreated controls. Continuous dosing condition, on the other hand, was significantly more effective in suppressing nitrite release starting at day 4 (*p* < 0.029 through day 18) compared to a single dose, keeping levels close to that of control. Of note is that both IL-1 and a single dose of IL-1Ra treatments resulted in significantly lower nitrite release when the synovium was present in C+S compared to that in C starting at day 2 (*p* < 0.0001) for IL-1 and at day 4 (*p* < 0.0001) for the single-dose IL-1Ra condition (Fig. 2c, d, Additional file 3: Figure S3). This is consistent with the trends in Fig. 2a,b where greater suppression of GAG loss was observed with the single dose of IL-1Ra in C+S co-culture compared to in C monoculture. Additionally, nitrite release spiked in C monoculture soon after IL-1Ra was removed from the culture, i.e., by day 4 whereas in C+S co-culture, the nitrite release spike was delayed until day 6 further highlighting the enhanced effectiveness of IL-1Ra in presence of synovium.

Treatment with IL-1 stimulated collagen loss from cartilage explants starting at day 10 compared to untreated controls (*p* < 0.0001), when about 30% GAGs were lost to media in both C and C+S conditions (Fig. 2e, f). Collagen loss continued to increase to about 40–50% of the total content in explant by day 24 when GAG loss had peaked to about 70%. A single dose of IL-1Ra significantly reduced collagen loss compared to IL-1-treated conditions beyond day 10 (*p* < 0.0001) in both C and C+S bringing levels down to that of untreated control, but at a later time point of day 24, the gap widened compared to control and became statistically significant (*p* < 0.0001) in C monoculture (but not in C+S co-culture) indicating time-dependent decrease in biological effectiveness. The continuous dose, however, suppressed collagen loss throughout the culture period. Note that the values for all C+S conditions are greater than the corresponding C conditions due to a larger contribution to collagen loss from the synovium tissue. Monocultures of cartilage and synovium revealed that synovium released 6.4× and 1.6× higher amounts of collagen than from cartilage by day 24 in control and IL-1, respectively (Additional file 4: Figure S4A and Additional file 4: Figure S4B). This also explains the slightly higher values of percent collagen loss observed with continuous dosing condition in C+S compared to in C.

### Continuous dose of IL-1Ra rescued chondrocyte metabolism and viability more effectively than a single dose

**Fig. 3:**
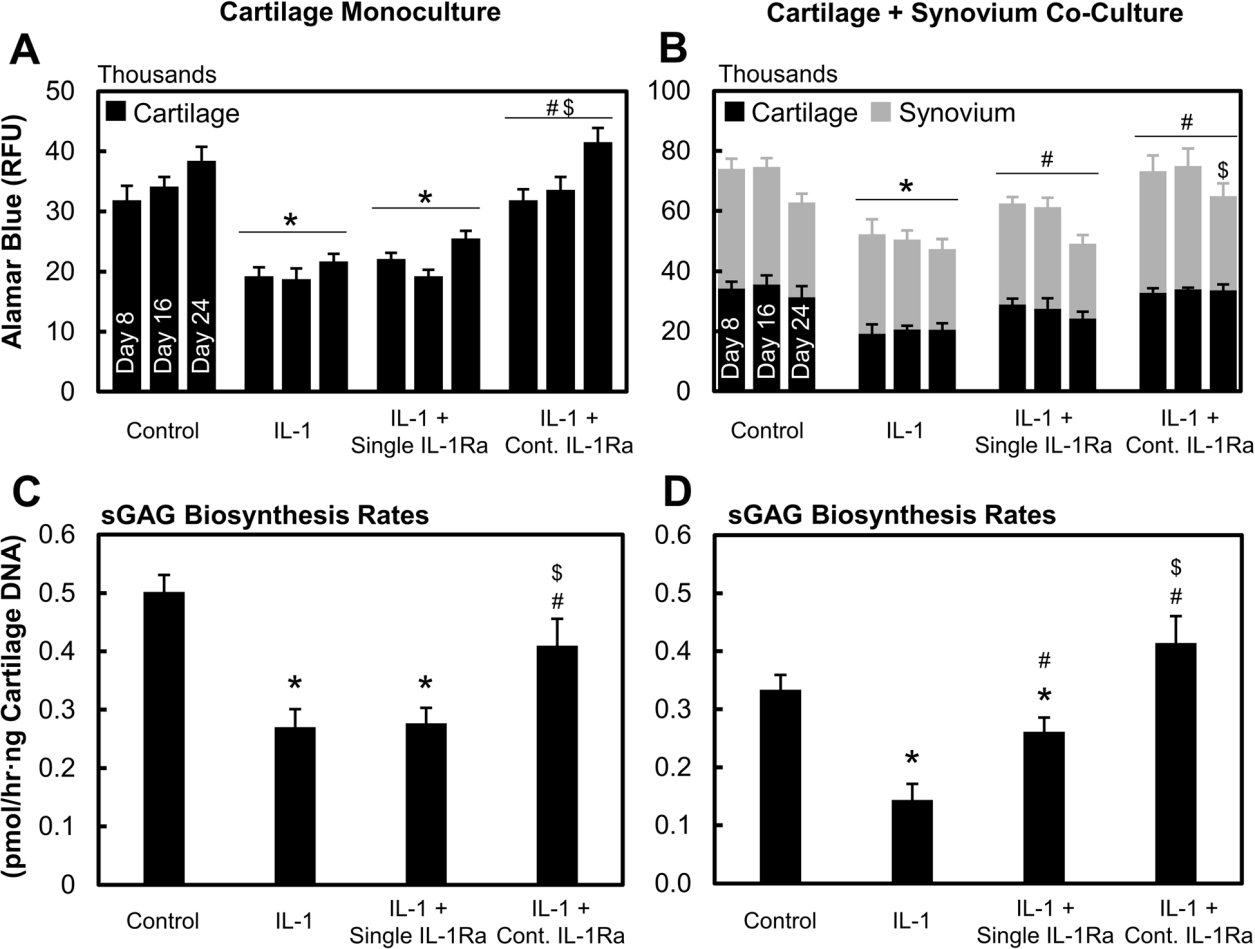
IL-1α-treated cultures administered with either a single or continuous (Cont.) dose of IL-1Ra. Cell metabolism of individual tissues in **a** cartilage monoculture and **b** cartilage + synovium co-culture. Rate of sGAG biosynthesis in cartilage tissue at day 16 in **c** cartilage monoculture and **d** cartilage + synovium co-culture. Data is presented as mean ± 95% confidence interval. * vs control, # vs IL-1, $ vs single-dose IL-1Ra, ^ vs corresponding cartilage monoculture (*p* < 0.05). Statistical markers are color coordinated with all bars

**Fig. 4:**
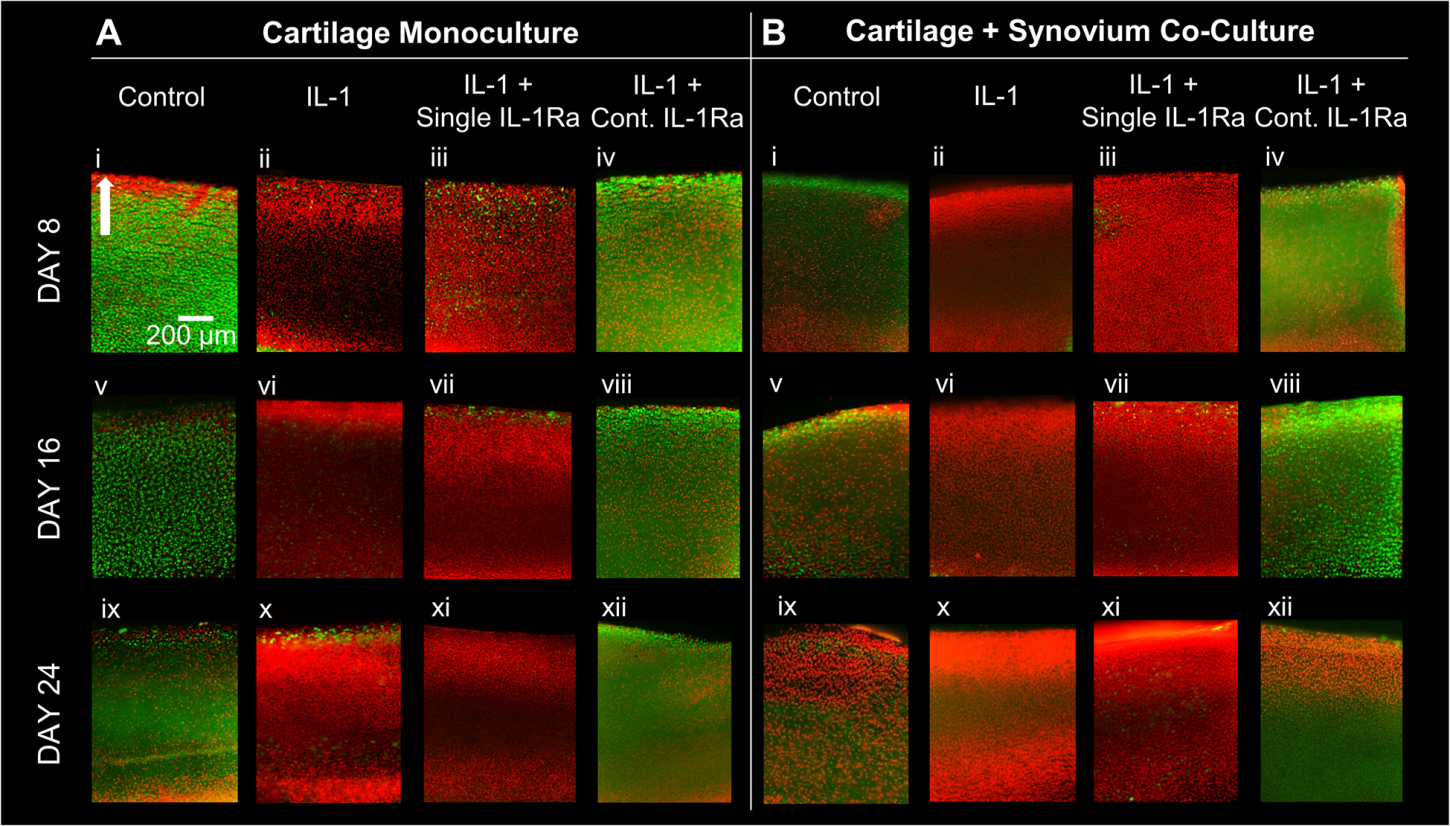
Chondrocyte viability images obtained on days 8, 16, and 24 in **a** cartilage monoculture and **b** cartilage + synovium co-culture treated with IL-1α and with a single or continuous (Cont.) dose of IL-1Ra. Viable cells shown in green, non-viable shown in red. Arrow indicates superficial layer of the tissue. Scale bar = 200 μm

In cartilage monoculture, a single dose of IL-1Ra was unable to rescue chondrocyte metabolic activity reduced by IL-1α treatment (Fig. 3a), while a continuous dose significantly rescued cell metabolism (*p* < 0.0001 compared to IL-1 or single-dose IL-1Ra) bringing them back to control levels. Similar trends were observed in C+S (Fig. 3b) except that the single dose of IL-1Ra showed rescuing efficacy at earlier time points through day 16, an effect that was not seen in cartilage monoculture. sGAG biosynthesis rates confirmed these results as a continuous dose of IL-1Ra significantly rescued IL-1-induced drop in sGAG synthesis rates (*p* < 0.0001) (Fig. 3c, d) while the single dose was not as effective. A single dose of IL-1Ra, however, restored aggrecan sGAG biosynthesis rates back to control levels in C+S co-culture (Fig. 3d) by day 16 but not in C monoculture (Fig. 3c), further highlighting the enhanced effectiveness of IL-1Ra in the presence of synovium tissue. The presence of synovium in C+S co-culture generally reduced biosynthesis rates compared to that in cartilage monoculture, which is also supported by the reduced chondrocyte viability observed in the presence of synovium in the untreated condition starting at day 16 (Fig. 4a, b).

A single dose of IL-1Ra was unable to rescue IL-1α-induced chondrocyte death in either C monoculture or C+S co-culture by day 8 (Fig. 4a, b). Continuous dose, however, significantly inhibited IL-1-induced cell death throughout the culture period of 24 days in both mono- and co-culture conditions. Note that some cell death in the superficial zone is typically observed in untreated control explants, depending on the location of harvesting along the joint. Also, excision of tissues from the joint using punches can also lead to cell death at the cut surfaces [23].

### Presence of synovium in C+S co-culture showed increased IL-4 levels in day 2 media

**Fig. 5:**
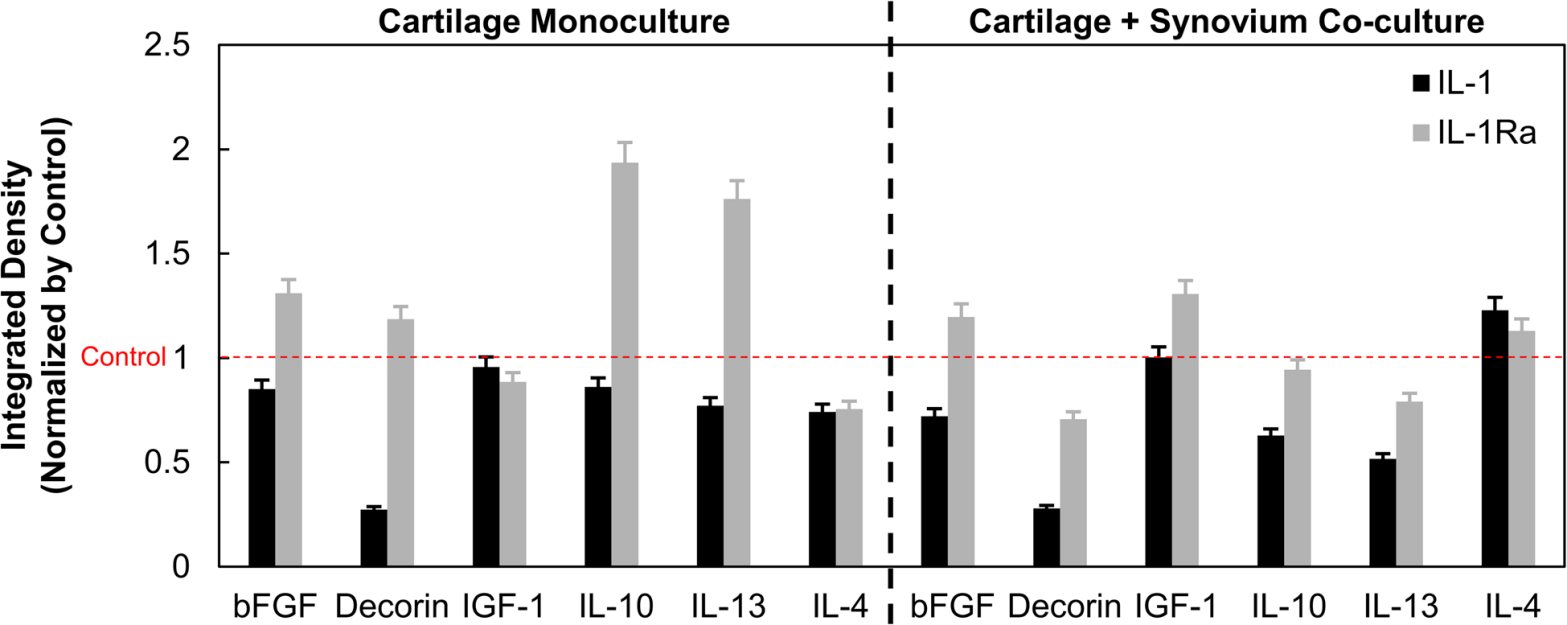
Integrated density measured using cytokine dot blot assay for IL-1- and IL-1Ra-treated condition media pooled from days 2 and 4 normalized by that of untreated control in both cartilage monoculture and cartilage + synovium co-culture

We used a bovine cytokine array kit to measure relative concentrations of anabolic and anti-inflammatory factors in day 2 condition media (Fig. 5). With IL-1 treatment, lower levels of pro-anabolic factors (e.g., basic fibroblast growth factor (bFGF), insulin-like growth factor-1 (IGF-1)) and anti-inflammatory cytokines (e.g., IL-4, IL-10, IL-13) were released into the media, suggesting reduced synthesis levels. IL-1Ra abolished IL-1-induced inhibition of bFGF, decorin, IL-10, and IL-13 syntheses, thereby increasing their release in the media. Generally, similar trends were observed in cartilage monoculture and C+S co-culture. Of interest in the present context is that the conditioned media from IL-1-treated C+S measured higher levels of IL-4 compared to that in C. IL-4 is known to synergize with IL-1 to enhance endogenous IL-1Ra production [4, 24–26], reducing nitrite synthesis in C+S at early time points. This, in the presence of exogenous IL-1Ra, can further enhance its role in suppressing IL-1-induced catabolism in C+S.

### Secretome data reveals significant crosstalk between cartilage and synovium tissues

**Fig. 6:**
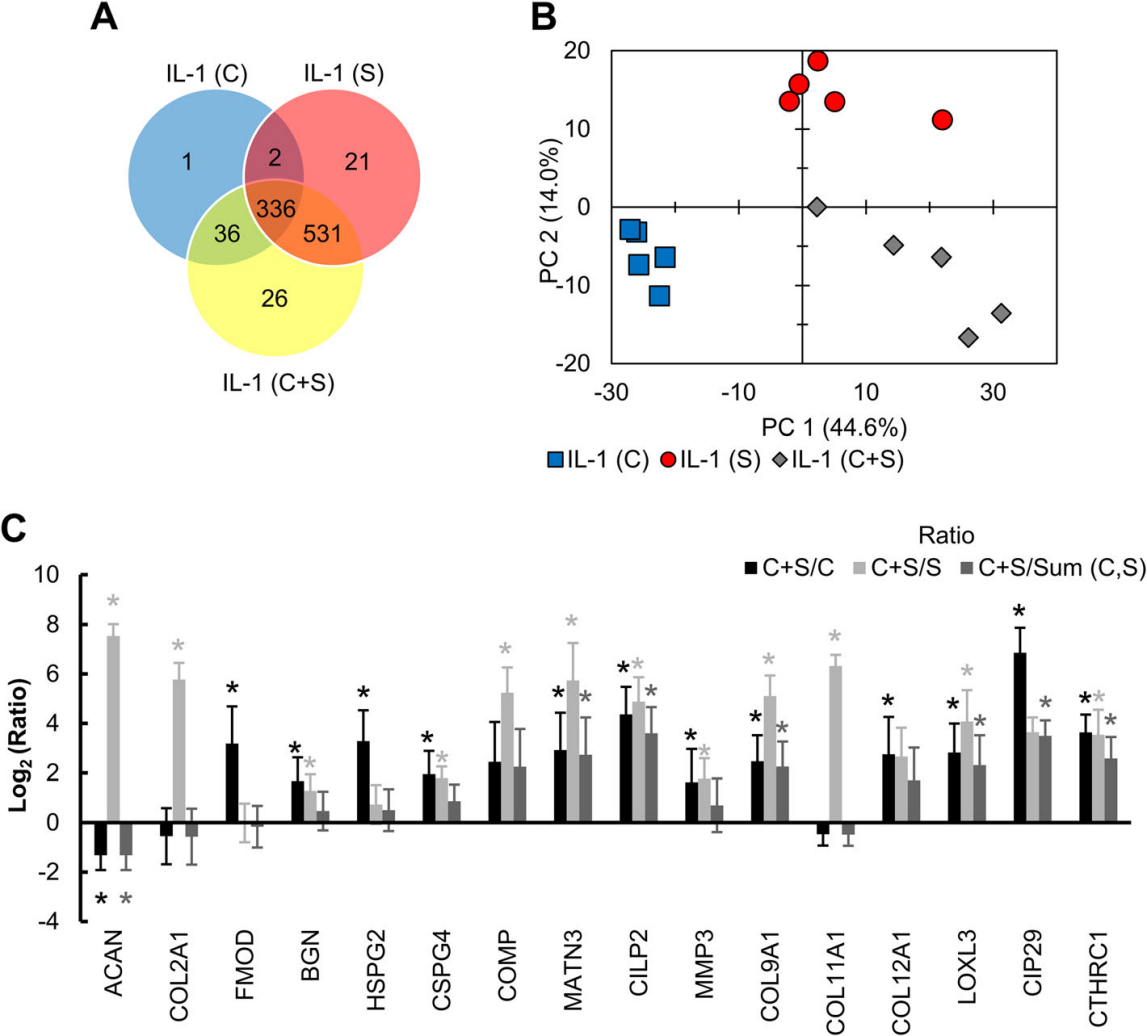
**a** Venn diagram showing distribution of 953 distinct proteins identified via mass spectrometry in day 2 conditioned media from IL-1-treated cartilage monoculture, synovium monoculture, and cartilage + synovium co-culture conditions. **b** Principal component analysis chart showing variances between samples based on proteins identified and their abundances. **c** Ratio of representative protein abundances identified in cartilage + synovium co-culture to either cartilage (C+S/C), synovium monoculture (C+S/S), or the sum of cartilage monoculture and synovium monoculture (C+S/Sum(C,S)) highlighting biological crosstalk. Data is presented as mean ± 95% confidence interval. * indicates significance between co-culture and denominator of ratio (*p* < 0.05). Statistical markers are color coordinated with all bars

To further analyze the role of synovium on cartilage health, secretome analysis was done using IL-1 conditioned day 2 media from C, S, and C+S conditions that identified 557 and 62 distinct proteins in C+S compared to C and S, respectively (Fig. 6a). One protein, connective tissue growth factor (CTGF), implicated in chondrogenesis and osteogenesis was found to be unique in IL-1 cartilage monoculture. A total of 26 unique proteins were identified in the C+S group (Table 1) out of which carbonic anhydrase III (CA3), transporter protein SEC31A, heme-binding protein 2 (HEBP2), and oxidative stress responsive 1 (OXSR1) are known to regulate oxygen tension and NO production.

**Table 1:** List of 26 distinct proteins uniquely identified in cartilage + synovium co-culture day 2 media

2-Iminobutanoate/2-iminopropanoate deaminase	LanC like 1
6-Phosphogluconolactonase	MAGE family member D2
Acetyl-CoA acetyltransferase 2	Oxidative stress responsive 1
Adducin 3	Papilin
Aminopeptidase	Peptidyl-prolyl cis-trans isomerase-like 1
Carbonic anhydrase 3	Poly(U)-binding-splicing factor PUF60
CCR4-NOT transcription complex subunit 3	Procollagen C-endopeptidase enhancer 2
General vesicular transport factor p115	Prostaglandin reductase 2
Heme-binding protein 2	SEC31 homolog A, COPII coat complex component
HSPC321 protein	Splicing factor 3A subunit 1
Interleukin enhancer binding factor 2	Spondin 2
Inverted formin, FH2 and WH2 domain containing	Thimet oligopeptidase
Isoamyl acetate-hydrolyzing esterase 1 homolog	Twisted gastrulation BMP signaling modulator 1

A 2-D principal component analysis chart based on protein abundance levels showed distinct clustering between the three groups revealing significant crosstalk across tissues (Fig. 6b). The abundance of matrilin-3 (MATN3), another protein that can mediate iNOS expression by regulating endogenous IL-1Ra production [27], was measured to be 8× and 52× higher in C+S media compared to C or S conditioned media respectively (Fig. 6c). Furthermore, among the proteins secreted predominantly by cartilage, aggrecan was 2.5× lower (*p* = 0.036), and fibromodulin (*p* = 0.004) and heparin sulfate proteoglycan (*p* = 0.005) were 9.8× higher in C+S media compared to C. Other representative proteins like ACAN, COMP, MATN3, CILP-2, Collagen-9, LOXL3, CIP29, and CTHRC1 (Fig. 6c, Table 2) were significantly under- or overexpressed in C+S media compared to the sum of protein abundance in C and S monocultures (Sum (C,S) in Fig. 6c), further corroborating the evidence of crosstalk.

**Table 2:** Proteins with expanded names plotted in Fig. 6c

Abbreviation	Protein
ACAN	Aggrecan Core Protein
COL2A1	Collagen alpha-1 (II)
FMOD	Fibromodulin
BGN	Biglycan
HSPG2	Heparan Sulfate Proteoglycan 2
CSPG4	Chondroitin Sulfate Proteoglycan 4
COMP	Cartilage Oligomeric Protein
MATN3	Matrilin 3
CILP2	Cartilage Intermediate Layer Protein 2
MMP3	Matrix Metalloproteinase 3
COL9A1	Collagen alpha-1 (IX)
COL11A1	Collagen alpha-1 (XI)
COL12A1	Collagen alpha-1 (XII)
LOXL3	Lysyl Oxidase Like 3
CIP29	Cytokine Induced Protein 29
CTHRC1	Collagen Triple Helix Repeat Containing 1

## Discussion

Since both cartilage and synovium tissues have IL-1R1 receptor sites, significant cellular crosstalk can impact the biological response to IL-1α and IL-1Ra. We investigate this by studying the time-dependent bio-activity of a single dose (mimicking single injection in vivo) and multiple doses (mimicking sustained drug concentration that an effective drug delivery system could enable with a single IA injection) of IL-1Ra in both in vitro monoculture of cartilage and co-culture of cartilage and synovium tissue explants. We first demonstrate that the presence of synovium in co-culture models enhances the beneficial effects of IL-1Ra in suppressing cytokine-induced catabolism. Additionally, our 24-day culture experiments reinforce that a single dose of IL-1Ra was ineffective and a sustained dose was necessary to significantly suppress IL-1α-induced catabolism in cartilage long term as demonstrated by enhanced suppression of GAG loss, NO synthesis, and rescued chondrocyte metabolism, viability, and GAG biosynthesis rates (Figs. 2, 3, and 4). This is consistent with the results of clinical trial NCT00110916, which evaluated the effects of one-time intra-articular injection of 150 mg IL-1Ra in patients with knee OA that suppressed pain only until day 4, with no changes in biomarker evaluation of cartilage degradation observed over a 1-month period; this was attributed to IL-1Ra’s short joint residence time and lack of cartilage targeting [5, 28]. Successful animal studies that led to this trial had used frequent, repeated dosing [6–8], which is clinically impractical and comes with increased risk of intra-articular infection. The limitations with current delivery options underscore the need for developing drug delivery strategies that can achieve sustained IL-1Ra concentrations inside the joint long term with a single IA dose. In a separate pilot study (NCT00332254), a single IL-1Ra injection within 4 weeks of anterior cruciate ligament (ACL) rupture, decreased synovial fluid levels of IL-1α that significantly reduced pain and improved function but only short term, suggesting that IL-1Ra-induced inhibition of IL-1 immediately after injury is a viable therapeutic option [13]. Currently, a phase 2 trial (NCT02930122) is underway evaluating the effectiveness of multiple IA injections of 150 mg IL-1Ra (Anakinra) within 15 days of an ACL tear in 14–33-year-old females [29]. In our study, a single dose of IL-1Ra had similar efficacy in suppressing collagen loss as the continuous dose; differences between the groups became significant only at day 24 (Fig. 2e, f). This suggests that there are advantages of early intervention, before the degenerative point of no return, which is marked by significant GAG loss and the onset of collagen loss [1]. A drug delivery system that can mimic the effects of a continuous (sustained) dose with a single administration of IL-1Ra can potentially enhance its therapeutic effects, both symptomatically and through disease modification while minimizing side effects associated with repetitive drug doses [30, 31]. Multiple intra-articular injections can result in high drug concentration in serum following clearance from the joint space via the lymphatics and vasculature; for some drugs, this could potentially cause systemic toxicity. Approaches utilizing electrostatic interactions to rapidly deliver IA-injected drugs (modified to possess optimal cationic charge) into the negatively charged cartilage in high concentrations before they exit from the joint space have been shown to be effective [32, 33].

In the veterinary field, autologous conditioned serum (ACS) containing high concentrations of IRAP (interleukin-1 receptor antagonist protein) has shown promise but with multiple doses [34]. ACS, when administered to OA-induced horse carpal joints every week for 5 weeks significantly reduced lameness, synovial membrane hyperplasia, gross cartilage fibrillation, and synovium hemorrhage [34]. Orthokine (Arthrex VetSystems), an IRAP treatment approved for veterinary use, when administered IA twice a week for three consecutive weeks in patients with radiographic knee OA, showed significant improvements in WOMAC and VAS scores compared to placebo at 7, 13, and 26 weeks [35]. The aforementioned studies highlight the current interest in IL-1 inhibition therapies for OA treatment.

Consistent with prior literature [9, 16, 36, 37], some of our data at later time points seem to suggest that the presence of synovium in co-culture models worsens cartilage health as shown by reduced chondrocyte metabolism, sGAG biosynthesis rates, and viability (Figs. 3 and 4). This is a limitation of the in vitro model where the excised synovium tissue creates a traumatic environment for chondrocytes through the release of soluble degradative and inflammatory factors. This is naturally not the case in a native joint environment, where the synovium (and other tissues) will at least strive to maintain joint homeostasis and might also offer protection to cartilage in response to a mechanical/chemical insult. In our study, the protective effects of synovium are highlighted through results from Figs. 2, 3, and 4 where a single dose of IL-1Ra suppressed IL-1-induced GAG loss, NO synthesis, and at a later time point also collagen loss, as well as restored cell metabolism significantly greater in C+S co-culture than in C monoculture. The enhanced effectiveness of IL-1Ra in the presence of synovium can be potentially attributed to the release of endogenous anti-inflammatory factors from synovium at early time points in response to cytokine challenge, as indicated by higher IL-4 levels measured in C+S co-cultures compared to those in C monocultures (Fig. 5). In vitro and in vivo studies have shown that regulatory cytokines IL-4, IL-10, and IL-13, which are produced by synovial macrophages and various subtypes of T cells, can synergize with IL-1β to enhance production of IL-1Ra and soluble IL-1 receptors from synoviocytes [25, 26, 38, 39], inhibiting MMP secretion and proteoglycan loss in cartilage. In addition to a direct decrease in the secretion of inflammatory cytokines, IL-4 has been shown to reduce IL-1-induced NO production in primary bovine chondrocytes [24] as well as in vivo in a mechanical instability-induced rat OA model [40]. Our experiments showed reduced NO release in C+S co-culture compared to C in presence of IL-1 with or without IL-1Ra. To ensure that NO reduction was not due to increased cell death with IL-1 treatment, we checked chondrocyte viability at early time points of days 2 and 4 (Additional file 5: Figure S5), which was similar to untreated control condition. Chondrocyte viability, however, decreased from days 8 through 24 in both C and C+S conditions (Fig. 4). This suggests that while nitrite production at later time points may be attributed to cell death, this is not the case at earlier time points. Furthermore, we confirmed that the amount of synovium used in co-culture did not reduce IL-1 availability for cartilage, which could also contribute to reduced nitrite production, as the presence of 25 mg and 10 mg synovium resulted in similar nitrite levels (Additional file 6: Figure S6). These findings suggest that upon being challenged with IL-1, synovium may release anti-inflammatory endogenous factors, which in the presence of exogenous IL-1Ra can enhance suppression of IL-1-induced catabolism. Treatment with IL-1Ra increased levels of other regulatory cytokines IL-10 and IL-13 in day 2 conditioned media of both C and C+S, as expected. However, the increase was more prominent in C than in C+S co-culture. The effects of anti-inflammatory cytokines are not uniform in our study and warrant deeper probing to understand their individual roles and relative importance.

The role of oxidative stress and its effect on NO production has been identified as a major player in OA inflammation [21, 41]. Oxidative stress occurs when the generation of reactive oxygen species (ROS) overcomes the scavenging abilities of antioxidants. NO is one of the primary ROS produced by chondrocytes and is known to be strongly stimulated by IL-1 [42, 43]. In OA, upregulated iNOS oxidizes guanidinyl nitrogens of arginine to anhydroxy arginine which then further oxidizes to citrulline releasing NO [42]. NO then diffuses out of the chondrocytes and contributes to inflammation and tissue destruction by enhancing production of MMPs, inhibiting synthesis of collagen and proteoglycans and promoting chondrocyte apoptosis [21, 22, 44]. Our results identified carbonic anhydrase III (CA3) as one of the 26 proteins unique to C+S co-culture; it is a Zn-containing intra-cellular metalloenzyme that has been shown to protect cells from oxidative stress due to its anti-oxidant properties; and under extreme oxidative stress conditions, it scavenges oxygen radicals that irreversibly oxidize its reactive cysteines [45]. This can explain the observed reduction in NO release at day 2 under IL-1 conditioned media from C+S condition compared to C monoculture. Moreover, studies have revealed higher levels of CA3 antibodies in rheumatoid arthritis patient serum, thereby downregulating anti-oxidant activity of CA3 and triggering an autoimmune response [46]. Other unique proteins identified in C+S media in the context of regulating NO synthesis include oxidative stress responsive 1 (OXSR1) and heme-binding protein 2 (HEBP2). HEBP2 binds with NO to stimulate its activation via the cyclic guanosine monophosphate (cGMP) signaling pathway [47, 48].

In addition, we also found that matrilin-3 (MATN3), an ECM adaptor protein that plays a structural role in forming filamentous matrix network by interacting with collagen fibrils and proteoglycans, was significantly elevated in C+S media compared to C or S. Recombinant human MATN3 protein has been shown to induce IL-1Ra gene expression in human primary chondrocytes and increase IL-1Ra levels in the presence of IL-1β, which led to enhanced expression of Col2 and ACAN and inhibited MMP-13, ADAMTS-4, and ADAMTS-5 [27]. Furthermore, increased MATN3 gene expression has been found in OA cartilage [49], which is thought to represent an attempt to inhibit IL-1-induced joint destruction. MATN3 may therefore be involved in the enhanced IL-1Ra therapeutic effect in C+S co-cultures relative to C monoculture.

Proteomics data also identified 26 unique proteins in IL-1α-treated day 2 media from C+S co-culture and the 2-D principal component analysis showed distinct clustering between C, S, and C+S groups, thereby further confirming significant crosstalk across tissues. Additionally, representative proteins like ACAN, COMP, MATN3, CILP-2, Collagen-9, LOXL3, CIP29, and CTHRC1 (Table 2) were found to be significantly under- or overexpressed in C+S media compared to the sum of protein abundance in C and S monoculture media. Note that in the absence of crosstalk, protein abundance in C+S would be expected to be equal to the sum of that in C and S. This crosstalk may be mediated by exosomes, since the proteomics analysis also identified the established exosome markers CD9, Heat shock 70 kDa protein 8 (HSC70), and Heat shock cognate 90 (HSC90) in conditioned media samples. Also, these markers were found in much higher abundance in synovium-containing samples (data not shown), suggesting synovium may be the primary source of exosomes in co-cultures. For example, a recent study showed that upon stimulation with IL-1β, exosomes from synovial fibroblasts induced osteoarthritic changes in chondrocytes [50].

The following points should be noted when comparing results of this study with other literature. We have used tissues from young bovine joints of tightly controlled age which minimizes animal- to-animal variability. It has been previously shown that in young tissue specimens, the animal-to-animal variation is the same as specimen-to-specimen variation within a single animal [1, 16, 17]. This was confirmed by our studies where three independent repeats showed the same trends. Additionally, previous work has validated that the effects of cytokine in immature bovine knee cartilage represent well the trends observed in adult human cartilage [1, 51], thus increasing our confidence in this approach. Healthy tissue was used to create a controlled PTOA model by IL-1 challenge and start IL-1Ra treatment at an early stage during the therapeutic “intervention window” and not wait until a later stage that can be highly variable. It is also noteworthy that the excision of synovial capsule and cartilage before culture traumatizes the tissue, and the cells may respond to this injury with an inflammatory phenotype [23, 37]. An untreated control condition, however, helps separate these effects. Our co-culture experiments contained multiple tissues in the same well with no barrier to separate them from physical contact, which could potentially introduce direct tissue interactions compared to co-cultures without any physical contact.

## Conclusions

This study shows that the effect of IL-1Ra in suppressing cytokine-induced catabolism is enhanced when cartilage and synovium are both present. When challenged with IL-1, synovium produces endogenous inhibitory factors as a measure of recovery, as demonstrated by enhanced IL-1Ra therapeutic effects in cartilage-synovium co-culture. These effects were associated with increased levels of the known anti-catabolic factors IL-4, carbonic anhydrase-3, and matrilin-3. Thus, to be meaningful, in vitro studies should consider the multifactorial nature of OA by using cartilage-synovium co-culture models instead of cartilage monocultures to assess its pathogenesis, progression, and response to therapeutics. Additionally, this study formally demonstrates that the chondroprotective effects of IL-1Ra on cartilage degeneration require sustained levels of the protein throughout the culture period. This underscores the unmet need for effective drug delivery strategies that can enhance IL-1Ra residence time inside the joint following its intra-articular administration and target both synovium-induced inflammation as well as chondrocytes throughout the full thickness of cartilage. Several methods for charge-based intra-cartilage [33, 52, 53] and joint depot drug delivery [54–56] are currently under development to address this need.

## Supplementary information

**Additional file 1: Figure S1.** Cumulative sGAG release as percentage of total sGAG content measured every 2 days in cartilage monoculture (C) and cartilage-synovium co-culture (C+S) treated with IL-1α ± Single dose IL-1Ra for 24 days. Data is presented as Mean ± 95% confidence interval. * indicates significant difference between co-culture and monoculture of respective treatment condition (*p*<0.05). Statistical markers are color coordinated with curves. All the data enclosed within similar markers is statistically significant. Continuous IL-1Ra conditions are not shown as the data are overlapping with their respective control conditions.

**Additional file 2: Figure S2A.** Nitrite released to media from cartilage (C) and synovium (S) tissue cultured individually and treated with IL-1α (2 ng/mL) for 8 days. B. Total tissue DNA content-normalized nitrite release from cartilage monoculture and cartilage-synovium co-culture in control and continuous dose IL-1Ra treatment conditions. Data is presented as Mean ± 95% confidence interval.

**Additional file 3: Figure S3.** Nitrite release normalized by DNA measured in media of cartilage monoculture (C) and cartilage-synovium co-culture (C+S) treated with IL-1α ± Single dose IL-1Ra for 24 days. Data is presented as Mean ± 95% confidence interval. * indicates significant difference between co-culture and monoculture of respective treatment condition (p<0.05). Statistical markers are color coordinated with curves. All the data enclosed within similar markers is statistically significant. Continuous IL-1Ra conditions are not shown as the data are overlapping with their respective control conditions.

**Additional file 4: Figure S4.** Monocultures of cartilage (C) and synovium (S) and co-culture of cartilage + synovium (C+S) treated with IL-1α for 24 days. Mean ± 95% confidence interval of A. cumulative collagen release as percentage of total collagen content and B. total collagen content remaining in cartilage, synovium and in media following 24 days of culture. * vs untreated control in respective culture, # vs untreated control C+S condition, $ vs IL-1 C+S condition, (p<0.05). Statistical markers are color coordinated with all curves and bars. All the data enclosed within similar markers is statistically significant.

**Additional file 5: Figure S5.** Chondrocyte viability images obtained from IL-1 treated cartilage slices on day 2 and 4 in A. cartilage monoculture and B. cartilage + synovium co-culture. Viable cells shown in green, non-viable shown in red. Arrow indicates superficial layer of tissue. Scale bar = 200 μm. C. Chondrocyte viability shown as percentage of total cells.

**Additional file 6: Figure S6.** Bovine cartilage (C) co-incubated with either 25 or 10 mg synovium (S) for 8 days and treated with IL-1. Mean ± 95% confidence interval of nitrite release measured in media every 2 days.

## Data Availability

All data generated or analyzed during this study are included in this published article and its supplementary information files.

## Abbreviations

AGC: Automatic gain control
bFGF: Basic fibroblast growth factor
CA3: Carbonic anhydrase III
CTGF: Connective tissue growth factor
DMEM: Dulbecco’s modified Eagle’s medium
DMMB: Dimethyl-methylene blue
DMOAD: Disease-modifying osteoarthritis drug
ECM: Extracellular matrix
FDA: Fluorescein diacetate
GAG: Glycosaminoglycan
HCD: High-energy collision-induced dissociation
HEBP2: Heme-binding protein 2
HEPES: 4-(2-Hydroxyethyl)-1-piperazineethanesulfonic acid
HSC70: Heat shock 70 kDa protein 8
HSC90: Heat shock cognate 90 protein
HSD: Honestly significant difference
IA: Intra-articular
IGF-1: Insulin-like growth factor-1
IL-1: Interleukin-1
IL-1R1: Interleukin-1 Receptor type 1
IL-1Ra: Interleukin-1 Receptor Antagonist
IL-1RAcp: Interleukin-1 Receptor accessory protein
iNOS: Inducible nitric oxide synthase
ITS: Insulin-transferrin-selenium
MATN3: Matrilin-3
MMP: Matrix metalloproteinase
MRI: Magnetic resonance imaging
MS: Mass spectrometry
NEAA: Non-essential amino acids
NF-κB: Nuclear factor kappa-light-chain-enhancer of activated B cells
NO: Nitric oxide
OA: Osteoarthritis
OXSR1: Oxidative stress responsive 1
PBS: Phosphate-buffered saline
PI: Propidium iodide
PTOA: Post-traumatic osteoarthritis
ROS: Reactive oxygen species
sGAG: Sulfated glycosaminoglycan
