# Supplementary figures and images for: Interleukin-1 receptor antagonist (IL-1Ra) is more effective in suppressing cytokine-induced catabolism in cartilage-synovium co-culture than in cartilage monoculture
Source: Arthritis Res Ther. 2019 Nov 13;21:238. doi: 10.1186/s13075-019-2003-y (PMC6854651; doi:10.1186/s13075-019-2003-y)

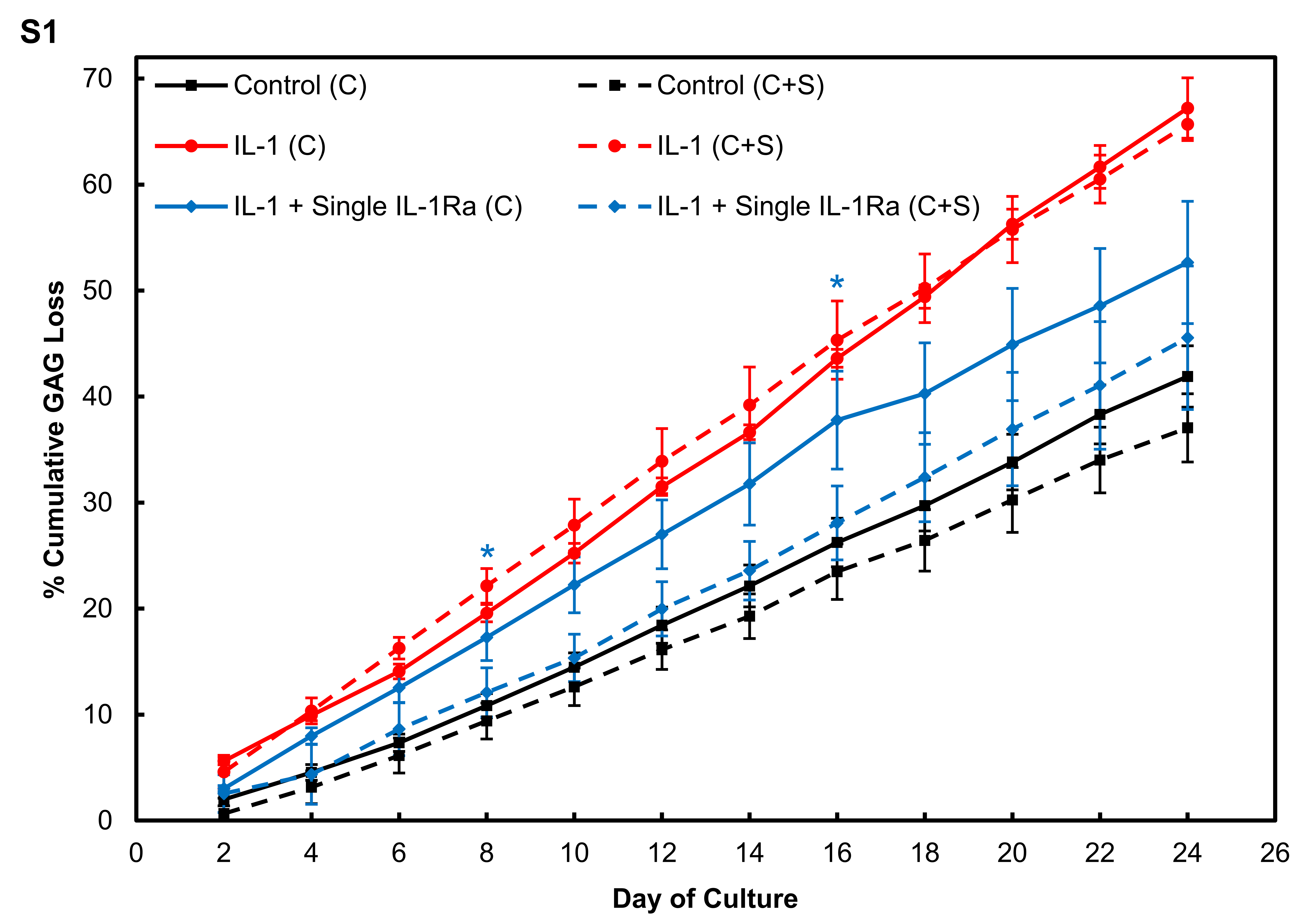

Supplement: Supplementary file 1 — Additional file 1: Figure S1. Cumulative sGAG release as percentage of total sGAG content measured every 2 days in cartilage monoculture (C) and cartilage-synovium co-culture (C+S) treated with IL-1α ± Single dose IL-1Ra for 24 days. Data is presented as Mean ± 95% confidence interval. * indicates significant difference between co-culture and monoculture of respective treatment condition (p<0.05). Statistical markers are color coordinated with curves. All the data enclosed within similar markers is statistically significant. Continuous IL-1Ra conditions are not shown as the data are overlapping with their respective control conditions. [file 13075_2019_2003_MOESM1_ESM.tif]

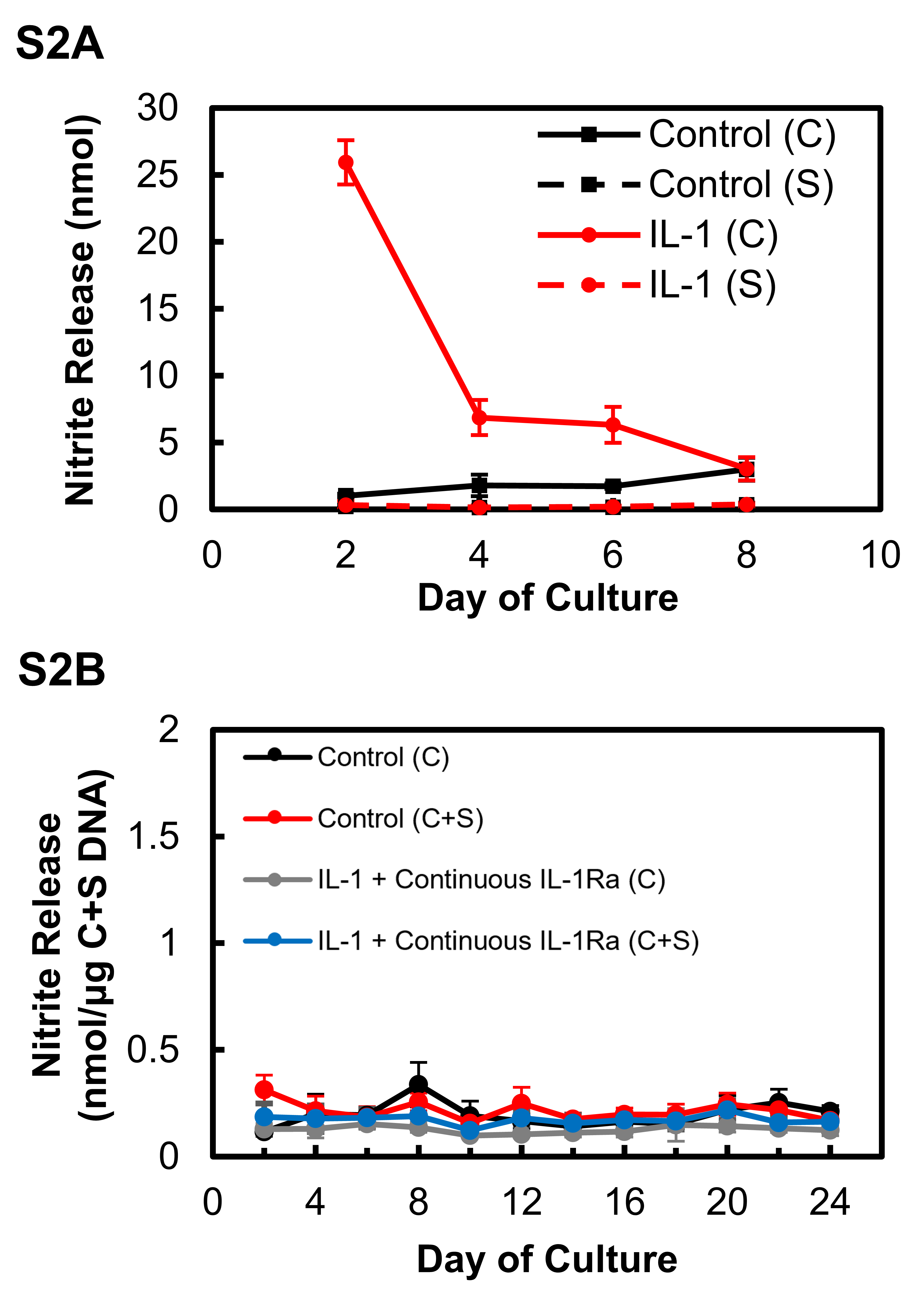

Supplement: Supplementary file 2 — Additional file 2: Figure S2A. Nitrite released to media from cartilage (C) and synovium (S) tissue cultured individually and treated with IL-1α (2 ng/mL) for 8 days. B. Total tissue DNA content-normalized nitrite release from cartilage monoculture and cartilage-synovium co-culture in control and continuous dose IL-1Ra treatment conditions. Data is presented as Mean ± 95% confidence interval. [file 13075_2019_2003_MOESM2_ESM.tif]

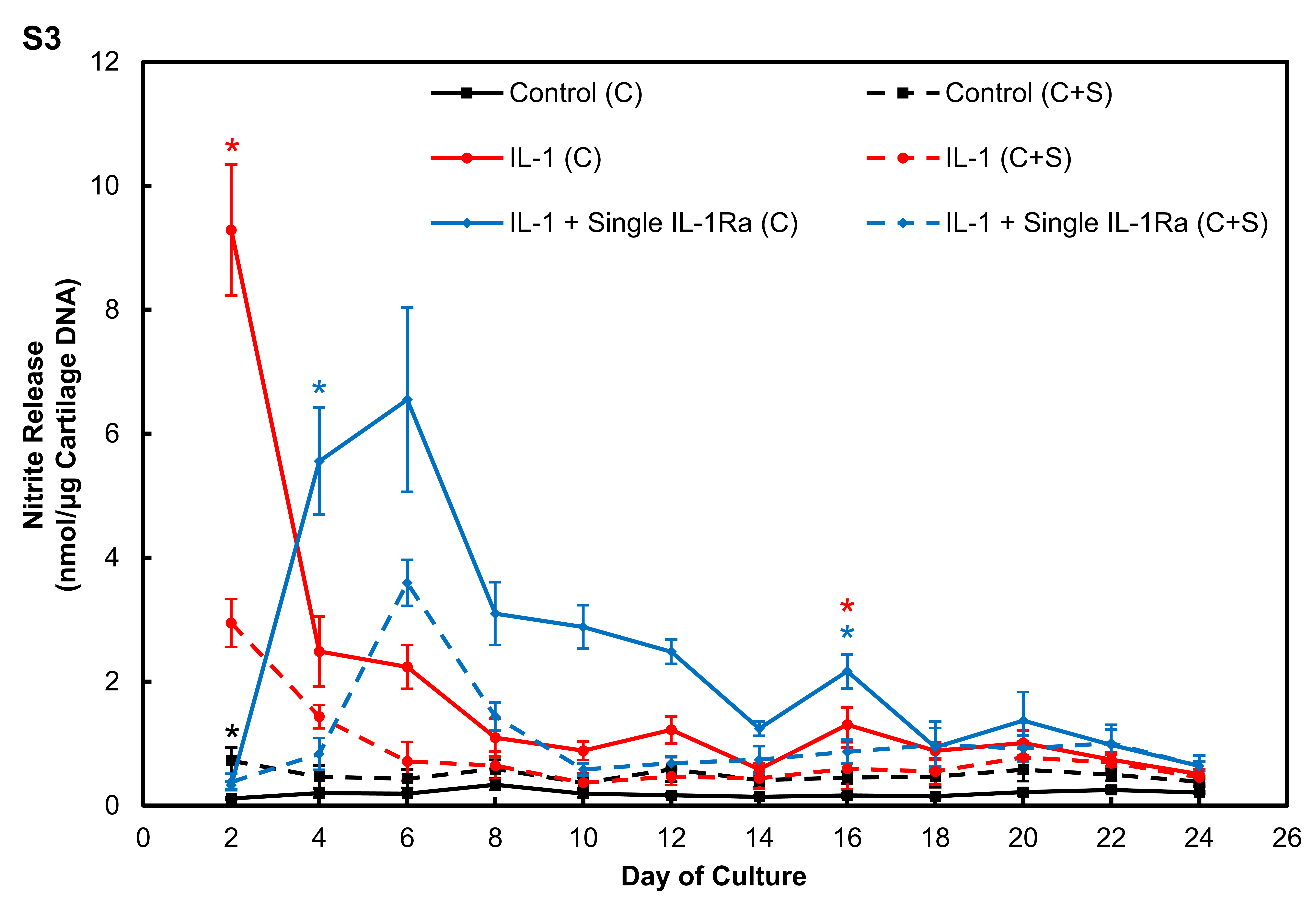

Supplement: Supplementary file 3 — Additional file 3: Figure S3. Nitrite release normalized by DNA measured in media of cartilage monoculture (C) and cartilage-synovium co-culture (C+S) treated with IL-1α ± Single dose IL-1Ra for 24 days. Data is presented as Mean ± 95% confidence interval. * indicates significant difference between co-culture and monoculture of respective treatment condition (p<0.05). Statistical markers are color coordinated with curves. All the data enclosed within similar markers is statistically significant. Continuous IL-1Ra conditions are not shown as the data are overlapping with their respective control conditions. [file 13075_2019_2003_MOESM3_ESM.tif]

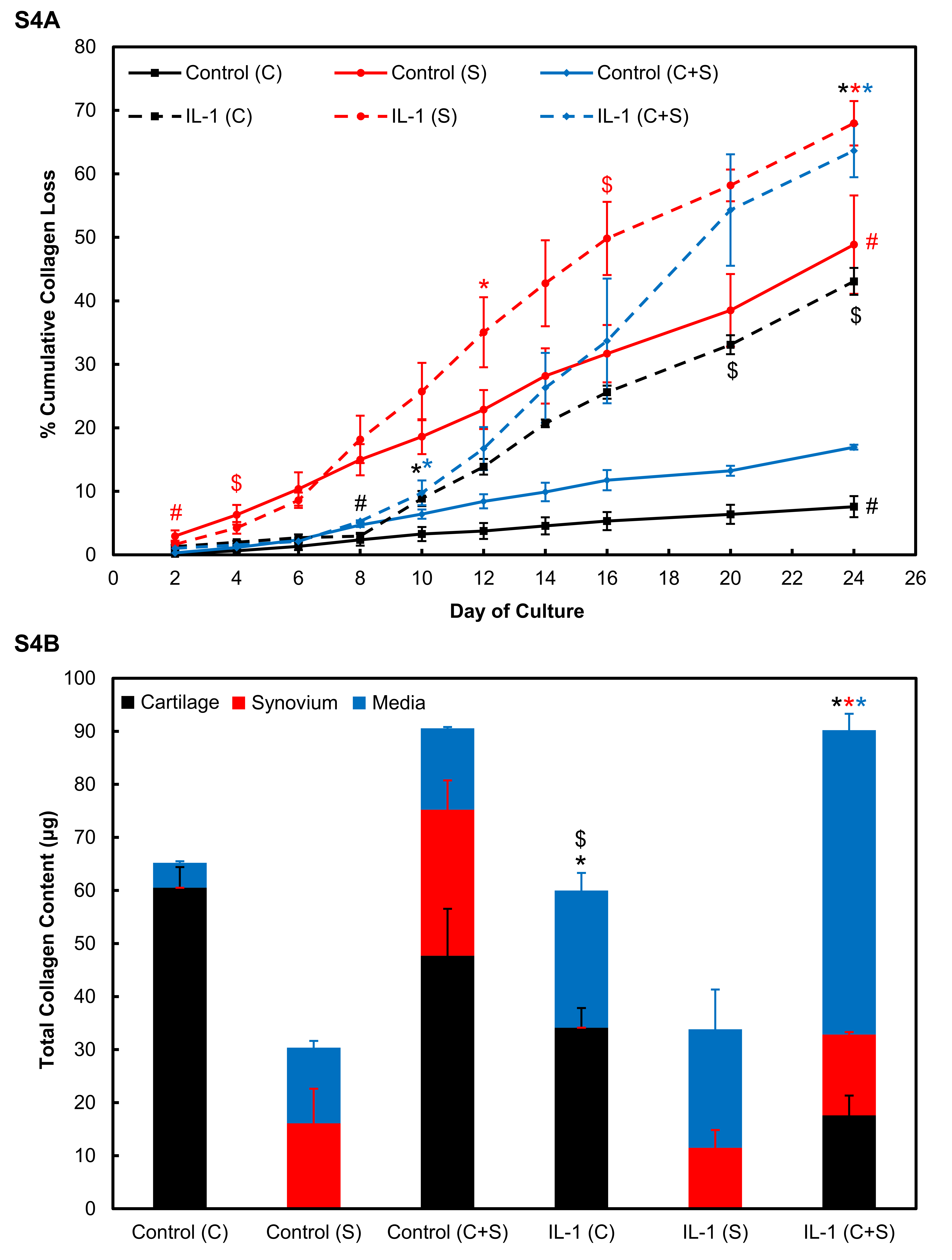

Supplement: Supplementary file 4 — Additional file 4: Figure S4. Monocultures of cartilage (C) and synovium (S) and co-culture of cartilage + synovium (C+S) treated with IL-1α for 24 days. Mean ± 95% confidence interval of A. cumulative collagen release as percentage of total collagen content and B. total collagen content remaining in cartilage, synovium and in media following 24 days of culture. * vs untreated control in respective culture, # vs untreated control C+S condition, $ vs IL-1 C+S condition, (p<0.05). Statistical markers are color coordinated with all curves and bars. All the data enclosed within similar markers is statistically significant. [file 13075_2019_2003_MOESM4_ESM.tif]

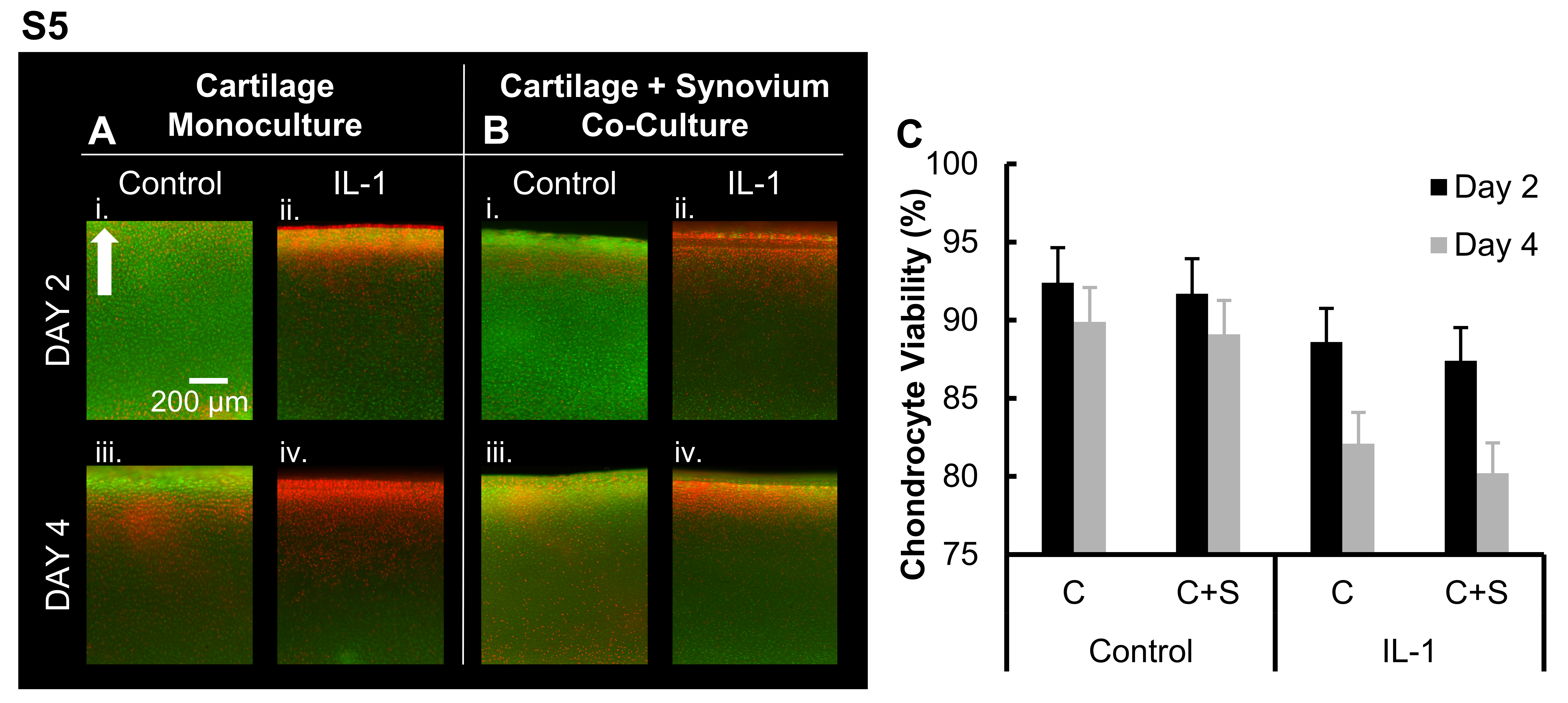

Supplement: Supplementary file 5 — Additional file 5: Figure S5. Chondrocyte viability images obtained from IL-1 treated cartilage slices on day 2 and 4 in A. cartilage monoculture and B. cartilage + synovium co-culture. Viable cells shown in green, non-viable shown in red. Arrow indicates superficial layer of tissue. Scale bar = 200 μm. C. Chondrocyte viability shown as percentage of total cells. [file 13075_2019_2003_MOESM5_ESM.tif]

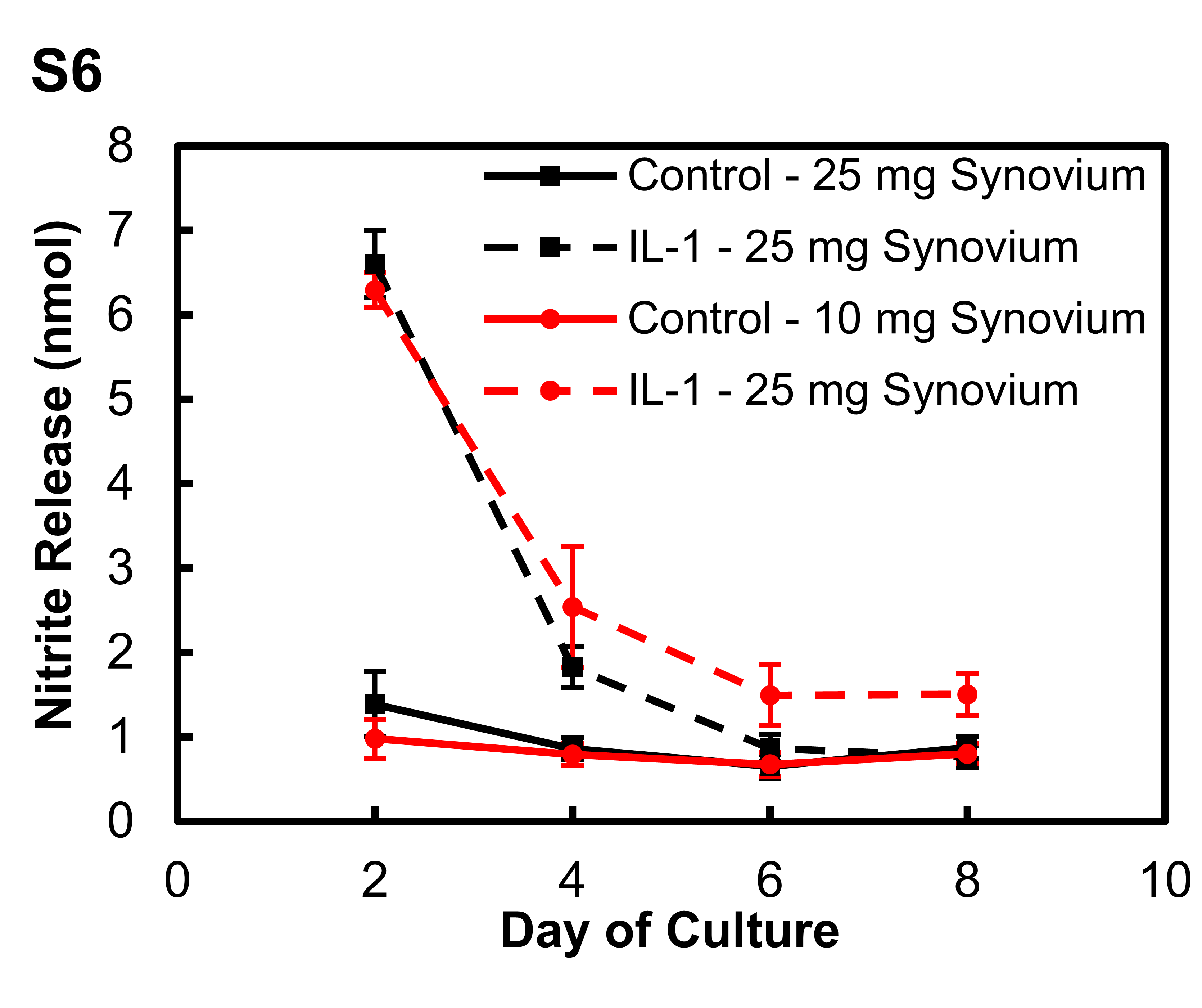

Supplement: Supplementary file 6 — Additional file 6: Figure S6. Bovine cartilage (C) co-incubated with either 25 or 10 mg synovium (S) for 8 days and treated with IL-1. Mean ± 95% confidence interval of nitrite release measured in media every 2 days. [file 13075_2019_2003_MOESM6_ESM.tif]
